# Current Status of Research on Losartan in Tumour Therapy

**DOI:** 10.1111/jcmm.70985

**Published:** 2026-01-04

**Authors:** Han Wang, Shuang Yuan, Hongjing Wang

**Affiliations:** ^1^ Department of Obstetrics and Gynecology, Key Laboratory of Birth Defects and Related Diseases of Women and Children of the Ministry of Education West China Second University Hospital, Sichuan University Chengdu China

**Keywords:** AT1R, cancer therapy, ECM remodelling, immune modulation, losartan, matrix stiffness, nanocarriers, RAS, TME, vascular normalisation

## Abstract

Losartan, a widely prescribed antihypertensive agent, has attracted growing interest as a potential adjuvant in cancer therapy due to its affordability, established safety profile and pleiotropic effects. Emerging preclinical evidence demonstrates that losartan can effectively modulate the tumour microenvironment (TME) by inhibiting transforming growth factor‐β (TGF‐β) signalling, reducing stromal stiffness and improving vascular perfusion. These changes are shown to enhance the delivery and efficacy of chemotherapeutic agents, an effect potentially amplified when combined with nanocarriers by augmenting the enhanced permeability and retention effect. Beyond TME remodelling, losartan has demonstrated anti‐tumour activity across various preclinical models, including those of pancreatic, breast and colorectal cancers. Mechanistically, angiotensin II type 1 receptor (AT1R) blockade is reported to modulate key downstream oncogenic pathways, including PI3K/AKT and YAP/TAZ, and to promote vascular normalisation via mechanisms that may include VEGF downregulation, thereby alleviating hypoxia and improving radiotherapy response. Furthermore, evidence suggests losartan remodels the tumour immune landscape by promoting CD8^+^ T and natural killer (NK) cell infiltration, reprogramming tumour‐associated macrophages (TAMs) and suppressing immunosuppressive cytokines. It also appears to inhibit epithelial‐mesenchymal transition (EMT) and metastasis‐related pathways, including CXCR4/SDF‐1α and matrix metalloproteinases (MMPs). These multifaceted mechanisms highlight its potential as a therapeutic adjuvant capable of overcoming stromal barriers, mitigating immune evasion and limiting metastatic dissemination. However, the translation of these compelling preclinical findings into clinical practice remains a major challenge. The promising preclinical data are tempered by variable efficacy across cancer types, a nascent clinical evidence base and unresolved questions regarding optimal patient selection and dosing. Clinical validation is still nascent, predominantly limited to early‐phase trials and critical parameters such as optimal dosing, treatment sequencing and long‐term safety in oncology patients await rigorous definition. This review synthesises the current mechanistic and translational research on losartan in solid tumours, aiming to clarify its anti‐cancer properties, explore its synergy with nano‐ and immune‐therapeutics, critically assess the associated challenges and identify key gaps and future directions for clinical application.

**Trial Registration:** ClinicalTrials.gov identifier: NCT01821729 and NCT03563248

## 1 Introduction

In 2022, approximately 4.82 million new cancer cases were projected in China, corresponding to an age‐standardised incidence rate of 208.58 per 100,000 individuals [1]. These statistics underscore the growing global burden of cancer and the urgent need for more effective therapeutic strategies. Despite considerable advances in oncology, conventional treatments—such as surgery, chemotherapy and radiotherapy—remain the primary therapeutic options for most tumours [2, 3, 4, 5]. However, limitations such as drug resistance, tumour recurrence and distant metastasis often result in unsatisfactory long‐term outcomes for many patients [6]. In recent years, increasing attention has been paid to the renin–angiotensin system (RAS), a complex signalling network whose aberrant activation contributes to multiple hallmarks of cancer, beyond its classical role in cardiovascular homeostasis [7, 8]. The key effector of RAS, angiotensin II (Ang II), is generated via an enzymatic cascade: liver‐derived angiotensinogen (AGT) is cleaved by renin into angiotensin I (Ang I), which is then converted to Ang II by angiotensin‐converting enzyme (ACE) primarily in the lungs [9].

Ang II exerts its biological effects predominantly through two G protein–coupled receptors: AT1R and angiotensin II type 2 receptor (AT2R). While AT1R is widely expressed in adult tissues and mediates classical RAS functions such as vasoconstriction, inflammation and fibrosis, AT2R is primarily active during fetal development and may antagonise AT1R signalling [10]. In oncology, the overactivated Ang II/AT1R axis is recognised as a key driver of tumour progression [7, 8, 11]. In preclinical findings, its signalling promotes a fibrotic and immunosuppressive TME and can directly stimulate cancer cell proliferation [12, 13, 14]. For instance, it directly stimulates cancer‐associated fibroblasts to deposit extracellular matrix (ECM) and contributes to the formation of abnormal, dysfunctional tumour vasculature, which impedes drug delivery [13, 14]. Conversely, the ACE2/Ang‐(1‐7)/MAS axis and AT2R signalling may counterbalance these oncogenic processes, although their mechanisms remain incompletely understood [15, 16].

Pharmacologic inhibition of the RAS has been extensively applied in the management of hypertension, heart failure and diabetic nephropathy using ACE inhibitors (ACEIs) and angiotensin receptor blockers (ARBs). Among these, losartan—the first ARB approved by the U.S. FDA—has demonstrated not only cardiovascular benefits but also potential anti‐tumour properties in a growing body of preclinical studies [17]. Compelling preclinical evidence demonstrates that by inhibiting TGF‐β signalling, losartan can potently remodel the TME, reducing ECM stiffness and collagen deposition while promoting tumour vascular normalisation; these changes can synergistically enhance perfusion and the intratumoral penetration of conventional chemotherapeutics [13, 18]. Specifically, in a murine model of non‐small cell lung cancer, losartan potentiated the antitumor efficacy of cisplatin, leading to a marked suppression of tumour growth [19]. Crucially, this stromal modulation strategy is particularly effective when combined with nanocarrier‐based drug delivery systems, as it overcomes the primary physical barriers that limit nanoparticle accumulation and distribution [18, 20].

Beyond TME modulation, losartan monotherapy exhibits direct anti‐tumour and metastasis‐suppressive effects in preclinical models of pancreatic, breast and colorectal cancers [12]. Importantly, by dismantling stromal barriers and reprogramming the immunosuppressive TME—e.g., increasing cytotoxic T cell infiltration—losartan has been shown to reverse resistance to immune checkpoint blockade therapy in models of breast and pancreatic cancer [21, 22]. Furthermore, combining losartan with radiotherapy in a HER2/neu‐positive orthotopic breast cancer model increased tumour control and inhibited lung metastases [23]. From a broader perspective, the reprogramming effect of RAS inhibitors on the tumour immune microenvironment is gaining increased scrutiny [11]. A pan‐cancer analysis suggests that the expression of RAS‐related genes is closely associated with patient prognosis and responses to immunotherapy, providing a solid rationale for their application in cancer immunology [24]. Despite compelling preclinical findings, the translational landscape for losartan is complex and punctuated by challenges. Promising early‐phase trials incorporating losartan into neoadjuvant therapy for pancreatic cancer have improved resection rates and survival but await validation in larger randomised trials [25] Additionally, the dual role of RAS in physiology and pathology necessitates a careful evaluation of potential side effects. For instance, hypertension is a common side effect of anti‐VEGF agents like bevacizumab, which could complicate its combination with losartan and underscores the critical need for predictive biomarkers to identify patient subsets most likely to benefit from this repurposed therapeutic strategy [26].

This review critically synthesises the current preclinical and translational evidence on losartan in cancer therapy, with a particular emphasis on its multifaceted mechanisms of TME remodelling and its potential to overcome therapeutic resistance across diverse cancer types. We explore its molecular mechanisms of action, its ability to remodel the TME and its synergistic potential with chemotherapy, radiotherapy and nanomedicine. We also highlight gaps in current knowledge and outline future research directions aimed at translating these findings into effective clinical strategies.

## 2 Pharmacological Profile and Emerging Oncological Relevance of Losartan

Losartan was the first non‐peptide AT1R antagonist approved for clinical use, establishing its role as the prototype of the ARB class [27]. Structurally, it shares a tetrazole–biphenyl scaffold with other ARBs, such as candesartan, irbesartan and valsartan [28]. A distinguishing feature of losartan is its hepatic biotransformation into an active metabolite, EXP‐3174, primarily via CYP2C9 [29]. This metabolite is 10–40 times more potent than the parent compound in AT1R blockade and has a significantly longer elimination half‐life [30]. Unlike most ARBs that act directly, losartan's antihypertensive efficacy depends largely on this metabolic activation, which accounts for a relatively small fraction of the administered dose. The parent compound has a short half‐life, moderate oral bioavailability and high plasma protein binding, necessitating once‐ or twice‐daily dosing. Additionally, its pharmacokinetics are significantly influenced by CYP2C9 genetic polymorphisms, which may affect EXP‐3174 exposure and therapeutic response [29, 31].

Losartan received FDA approval in 1995 for the treatment of essential hypertension and the reduction of stroke risk in patients with left ventricular hypertrophy [32]. Its indications were later expanded to include diabetic nephropathy in patients with type 2 diabetes, owing to its established reno‐protective effects [33, 34]. Clinical studies and meta‐analyses have consistently demonstrated its favourable safety and tolerability profile [35, 36]. Common adverse events are generally mild, and compared with ACEIs, losartan is associated with a significantly lower incidence of cough and angioedema. This established safety record has provided a strong rationale for investigating its repurposing potential in oncology. Beyond its conventional indications, growing attention has been directed toward losartan's potential antitumor effects. In a large population‐based case–control study, Chang et al. reported in diabetic patients that losartan use correlated with a 22% reduction in cancer risk (OR = 0.78; 95% CI = 0.63 to 0.97) [37]. The antitumor mechanisms of losartan are multifaceted and extend beyond simple receptor blockade. Preclinical studies delineate that it attenuates TGF‐β signalling, leading to a significant reduction in ECM stiffness and collagen deposition. This mechano‐therapeutic effect, particularly evident in breast cancer models, potently remodels the TME to enhance treatment efficacy [38]. Furthermore, losartan can induce direct anti‐proliferative and pro‐apoptotic effects in cancer cells, as seen in gastric cancer cell lines where it scavenges reactive oxygen species and inhibits cell growth [39].

A growing body of evidence has supported the antitumor activity of losartan across various solid malignancies, demonstrating its applicability in diverse contexts. In pancreatic ductal adenocarcinoma (PDAC), losartan reduces ECM accumulation and normalises tumour vasculature, improving the efficacy of chemotherapy and radiotherapy [13, 40]. A phase II/single‐arm (NCT01821729) demonstrated that adding losartan to FOLFIRINOX and chemoradiation was well tolerated and associated with an increased rate of R0 resection (~60%), with several ongoing phase II trials (e.g., NCT05861336) underway. In breast cancer, its role as a mechanotherapeutic adjuvant is well established, suppressing metastasis by depleting collagen I and enhancing chemotherapy penetration [38, 41]. In non‐small cell lung cancer (NSCLC), losartan significantly potentiated the antitumor efficacy of cisplatin in murine models, highlighting its strong chemosensitizing potential [19]. In hepatocellular carcinoma (HCC), it not only ameliorates liver fibrosis but also sensitises tumour cells to lenvatinib and enhances infiltration by effector T cells [42, 43]. Losartan‐based nanocomposite hydrogels represent a promising approach to overcoming therapeutic resistance, providing controlled release to continuously remodel the tumour mechanical microenvironment and effectively reverse chemo‐immunotherapy resistance [44]. Moreover, angiotensin receptor blockers like losartan can attack immunologically ‘armoured’ and ‘cold’ tumours by modulating myeloid cells and immune checkpoint expression, thereby potentially enhancing the response to immune checkpoint blockade [45]. Beyond conventional therapies, losartan has been combined with innovative modalities such as photothermal therapy, where its combination with platinum nanoparticles induced immunogenic cell death in neuroblastoma models, showcasing its versatility [46]. In ovarian cancer, losartan overcomes resistance to chemo‐immunotherapy by suppressing IGF‐1 signalling and rewiring the tumour‐immune microenvironment [47].

The ongoing clinical investigation of losartan, particularly in PDAC, positions it as a frontrunner in stromal‐targeting strategies. Its well‐established safety profile, low cost and multimodal mechanisms of action offer distinct advantages over many novel experimental TME‐modulating agents such as specific anti‐fibrotic monoclonal antibodies, though direct head‐to‐head comparative efficacy data are still needed. These findings provide compelling support for the further clinical development of losartan as a versatile TME‐modulating agent in translational oncology. However, it is crucial to acknowledge that its efficacy as a monotherapy is likely limited, and its success is predominantly envisioned within rational combination strategies [13, 18, 19]. Furthermore, the reliance on metabolic activation and the impact of CYP2C9 polymorphisms introduce a layer of pharmacokinetic variability that must be considered in oncology dosing regimens, which may differ from its use in hypertension [29, 30, 31].

## 3 TME Modulation by Losartan

### 3.1 Inhibition of TGF‐β Signalling and ECM Remodelling

**FIGURE 1:**
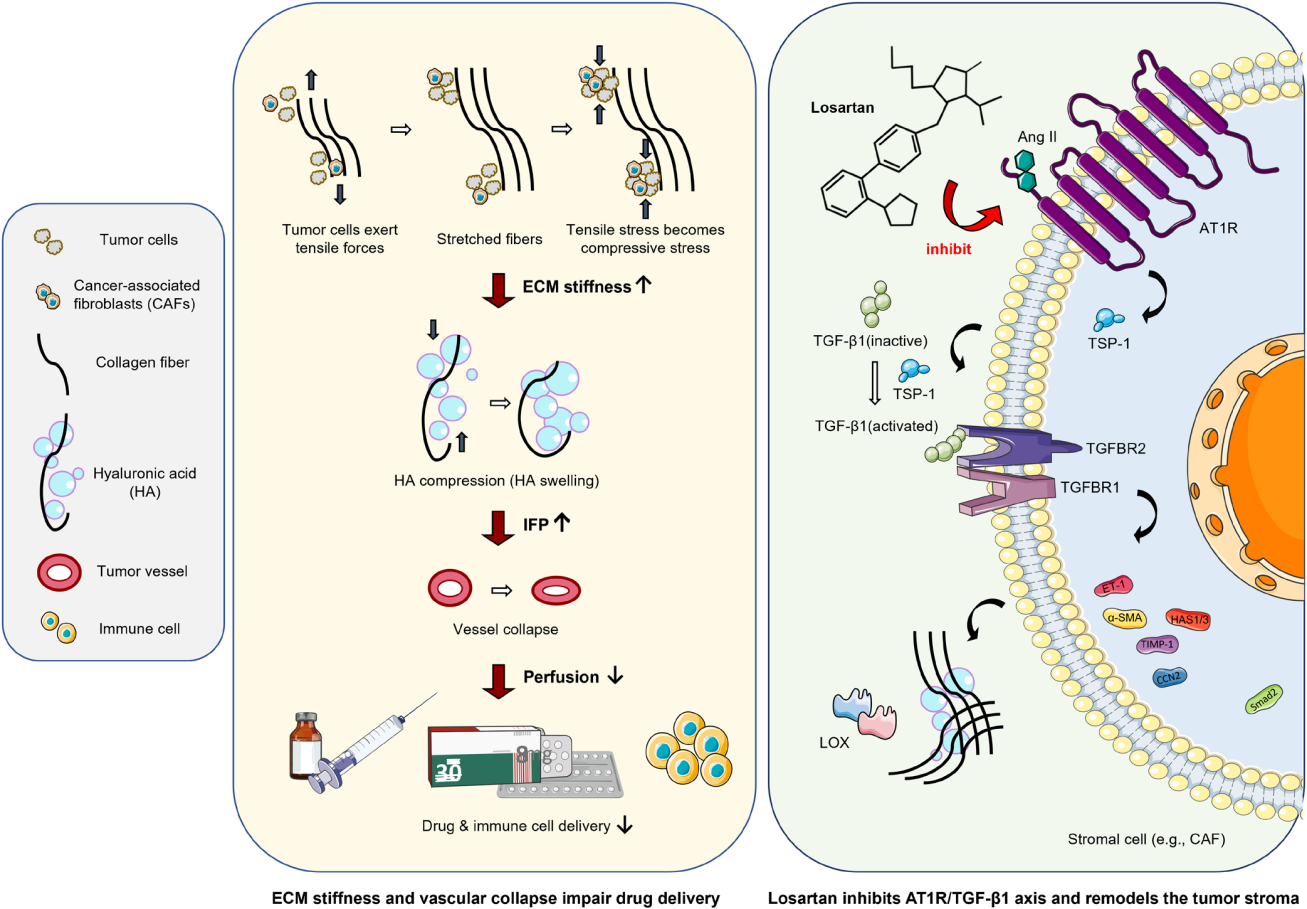
Losartan restores perfusion via AT1R/TGF‐β1 blockade and ECM remodelling. The schematic representations of drugs and cells in this figure were adapted from SMART Servier Medical Art, licensed under CC BY 3.0.

In collagen‐dense tumours, both malignant and stromal cells generate contractile forces that deform the surrounding ECM, particularly collagen fibres. This biomechanical tension accumulates within the matrix, increasing its stiffness. As tensile stress is gradually converted into compressive forces—particularly within glycosaminoglycan‐rich stroma—hyaluronic acid (HA) counteracts compression via electrostatic repulsion and water retention. Once HA reaches its deformation threshold, the resulting interstitial pressure is redirected toward adjacent vasculature, leading to vessel collapse, impaired perfusion and severely compromised drug delivery (Figure 1) [13, 48, 49, 50].

Although the RAS is traditionally recognised for its role in cardiovascular regulation, it also exerts critical local functions within the TME. In vivo and in vitro tumour models have been instrumental in validating AT1R as a key therapeutic target by elucidating its role in promoting a desmoplastic, fibrotic and immunosuppressive tumour microenvironment [51, 52, 53, 54]. Losartan, a selective AT1R antagonist, mitigates matrix remodelling by downregulating thrombospondin‐1 (TSP‐1), a key upstream activator of TGF‐β. Notably, losartan suppresses TGF‐β1 activation without reducing its total expression. Downstream, losartan inhibits several profibrotic mediators regulated by the AT1R/TGF‐β axis—including α‐smooth muscle actin (α‐SMA), integrins β3/β6, hyaluronan synthases (HAS1/3), endothelin‐1 (ET‐1), connective tissue growth factor (CCN2), Smad2 and TIMP‐1—ultimately decreasing collagen I/III and HA synthesis (Figure 1) [13, 18, 55, 56, 57].

Collectively, these molecular effects reduce ECM density and stiffness, lower interstitial fluid pressure (IFP) and markedly enhance the intratumoral delivery of chemotherapeutics, monoclonal antibodies and nanoparticle‐based agents. Notably, this stromal remodelling strategy is not exclusive to losartan. For instance, enzymatic degradation of HA with PEGylated hyaluronidase (PEGPH20) has also been shown to improve paclitaxel distribution and efficacy in preclinical models [58]. However, losartan's upstream targeting of the AT1R/TGF‐β axis offers a broader mechanism that concurrently addresses collagen deposition and HA synthesis. In HCC, losartan enhances T cell infiltration and suppresses TGF‐β signalling, mechanistically by inhibiting hepatic stellate cell (HSC) activation and reducing cancer‐associated fibroblast (CAF) accumulation [43]. This AT1R‐driven antifibrotic mechanism extends to PDAC, where aberrant RAS activation facilitates desmoplasia through stellate cell‐CAF interactions, exacerbating ECM deposition, vascular compression and solid stress [13, 18]. Direct evidence from a PDAC model shows that losartan treatment (20 mg·kg^−1^·day^−1^) decreased collagen I by 50%, which enhanced the intratumoral distribution and extravascular penetration of intravenously injected nanoparticles, ultimately leading to a significantly enhanced antitumor efficacy of nanotherapeutics [18].

CAFs are key mediators of ECM remodelling and therapeutic resistance [59]. They produce abundant fibrillar collagen and promote lysyl oxidase (LOX)‐mediated collagen crosslinking, increasing matrix stiffness and impairing drug delivery, particularly in myofibroblastic CAF subsets such as those in head and neck cancers, where NOX4/ROS‐dependent activation is involved. In addition, CAFs secrete immunomodulatory cytokines, including TGF‐β and IL‐6, and proangiogenic factors such as VEGF and bFGF, thereby fostering a tumour‐supportive microenvironment [60, 61]. They also recruit regulatory T cells (Tregs) and myeloid‐derived suppressor cells (MDSCs) through CXCL12 secretion, fostering an immune‐excluded and fibrotic niche. TGF‐β signalling further amplifies these effects by driving CAF differentiation, EMT and chemoresistance [43, 62]. Notably, remodelling this fibrotic and immunosuppressive TME, for example, by depleting CAFs, has been demonstrated to sensitise breast tumours to anti‐PD‐L1 immunotherapy, highlighting the critical link between stromal reprogramming and response to immune checkpoint blockade [63]. In ovarian cancer, losartan has been shown to downregulate ET‐1 in a dose‐dependent manner, inhibit α‐SMA^+^ fibroblast activation and induce miR‐133 as a downstream effector of TGF‐β suppression. This mechanistic underpinning is supported by clinical data, where ARB use was associated with a 45% reduction in the hazard of death (HR = 0.55) and a 30‐month extension in median overall survival [64]. However, overexpression of miR‐133 in tumour cells alone is insufficient to eliminate the fibrotic stroma, underscoring the importance of concurrently targeting stromal elements such as CAFs and TAMs [20, 64].

More recently, the antifibrotic and TME‐modulating effects of losartan have been extended to additional tumour types. In NSCLC, losartan suppresses EMT by upregulating E‐cadherin and downregulating vimentin, thereby enhancing cisplatin sensitivity both in vitro and in vivo [19]. In gastric cancer, losartan improved the efficacy of ^64^Cu‐trastuzumab radioimmunotherapy by remodelling the ECM and reducing IFP. Losartan treatment (40 mg/kg) significantly reduced IFP by 60% and enhanced trastuzumab accumulation by twofold, which contributed to a prolonged median survival of 37.5 days compared to 30.5 days in the control group. These effects were mechanistically linked to the downregulation of TGF‐β1, COL13A1 and SERPINE1, as well as upregulation of MMP2 [65]. The pursuit of anti‐fibrotic agents in oncology is an active field. The drug pirfenidone, for example, also exerts antitumor effects by targeting TGF‐β, as shown in renal cell carcinoma and colorectal cancer models, where it modifies the immunosuppressive TME and inhibits tumour progression [66, 67]. Interestingly, pirfenidone's effects are partially mediated through inhibition of MUC1 bioactivation, indicating a mechanism distinct from losartan's AT1R‐centric action [68]. Additionally, in vestibular schwannoma (VS), retrospective clinical data suggest that ARB use—including losartan—may be associated with improved hearing preservation, possibly via suppression of inflammation and fibrosis within neural tumours [69].

Taken together, these findings reinforce losartan's role as a potent TME‐normalising agent. Through inhibition of AT1R–TGF‐β signalling and depletion of activated CAFs, losartan alleviates matrix stiffness, decompresses vasculature and facilitates drug penetration. Its mechanism, targeting a specific receptor pathway (AT1R) with a proven safety record, presents a distinct approach compared to other strategies like enzymatic HA degradation or broad‐spectrum anti‐fibrotic. This antifibrotic mechanism has been validated in diverse solid tumour models—including PDAC, HCC, breast, ovarian, lung, gastric cancers and schwannomas—highlighting its translational promise as an adjuvant therapy in oncology. However, despite these compelling findings, the application of losartan and other anti‐fibrotic agents faces several challenges. The heterogeneity of CAF populations and the dynamic nature of the ECM mean that therapeutic responses can be variable and context‐dependent [70, 71]. For instance, targeting specific CAF subpopulations may trigger compensatory remodelling of the TME and yield unintended consequences, such as accelerated tumour progression, highlighting the complexity of stromal intervention [72]. Additionally, the optimal timing and sequencing of losartan with other treatment modalities remain to be fully elucidated in clinical settings [13, 73, 74].

### 3.2 Vascular Normalisation and Improved Drug Perfusion

**FIGURE 2:**
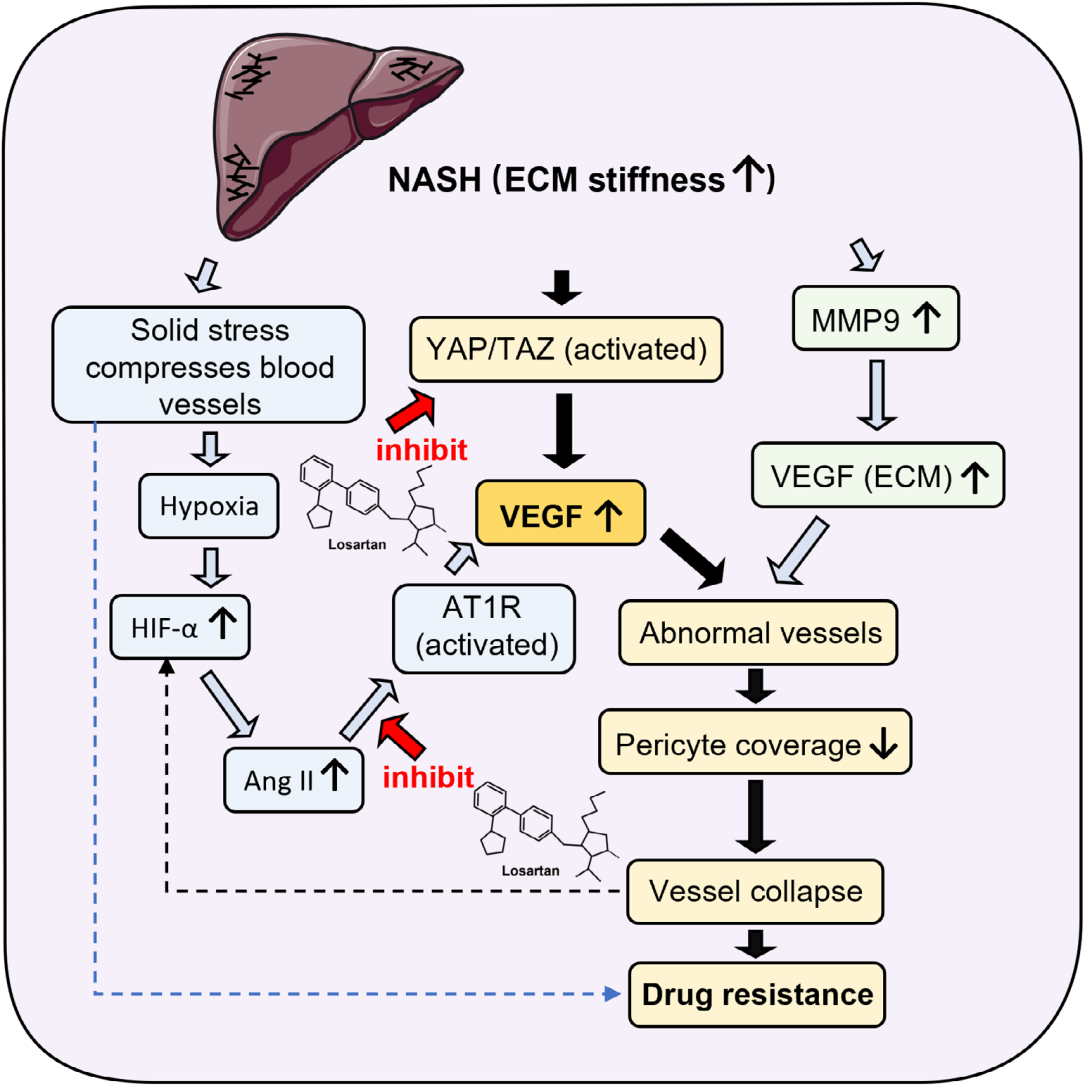
Losartan‐mediated vascular normalisation via AT1R and YAP/TAZ inhibition. The schematic representation of the liver in this figure was adapted from SMART Servier Medical Art, licensed under CC BY 3.0.

HCC frequently develops in fibrotic livers, particularly in the setting of non‐alcoholic steatohepatitis (NASH). In HCC, increased ECM stiffness is strongly associated with pathological angiogenesis. This aberrant vascular remodelling is mediated, at least in part, by YAP/TAZ activation in endothelial cells via two interrelated mechanisms: one is direct mechano‐transduction through integrin‐F‐actin signalling in response to matrix stiffening, and the other is YAP‐dependent stabilisation of Hypoxia‐inducible factor 1‐alpha (HIF‐1α) and subsequent amplification of the HIF‐1α/VEGF pathway [75, 76, 77]. Tumour endothelial cell‐derived MMP9 further contributes to neo‐vascularisation and metastasis by mobilising VEGF from ECM stores [78]. Simultaneously, excessive ECM deposition induces solid stress that compresses intratumoral vessels, reduces perfusion and hinders effective drug delivery (Figure 2) [13, 62].

Vascular dysfunction exacerbates intratumoral hypoxia and elevates local Ang II levels, which activate AT1R signalling and further augment VEGF‐driven angiogenesis. However, the induced neo‐vessels are frequently abnormal and fragile, prone to collapse and thereby paradoxically worsening hypoxia and therapeutic resistance [13, 62, 79]. In this context, losartan acts as a vascular modulator whose dominant benefits are tumour‐type dependent. In multiple solid‐tumour models, ARB treatment has been reported to decrease VEGF expression and to normalise aberrant vasculature, thereby improving perfusion and drug delivery; these vascular‐normalising effects have been proposed as a mechanism for enhanced chemotherapy efficacy [13, 62, 79]. In SPARC‐null PDAC models, the reduction in micro‐vessel density after losartan treatment is not linked to decreased VEGF mRNA, but is instead a consequence of stromal remodelling and mechanical decompression. This physical relief of vascular compression enhances drug penetration, underscoring stromal modulation as losartan's principal mechanism in PDAC [13, 80]. In HCC models, losartan has been shown to suppress Ang II–induced VEGF expression and to sensitise tumour cells to the multi‐kinase inhibitor lenvatinib, suggesting a role in overcoming autocrine VEGF‐mediated resistance [42]. In neurofibromatosis type 2 (NF2) schwannoma models, losartan improved vessel integrity and pericyte coverage without major changes in VEGF transcript levels or overall vascular density, pointing to a stabilisation of microvascular architecture rather than classical anti‐angiogenesis [81].

Beyond these examples, losartan's vascular effects manifest heterogeneously across tumour types. In colorectal and mammary tumour models, AT1R blockade inhibited tumour growth, suggesting potential anti‐angiogenic and vessel‐normalising activities that warrant further investigation [82, 83]. In ovarian cancer models, losartan treatment enhanced chemotherapy efficacy and reduced malignant ascites, an effect attributable to stromal normalisation and improved intratumoral distribution of cytotoxic agents [64]. Studies on metastatic lesions further indicate that decreasing matrix stiffness and decompressing vessels can improve responses to anti‐VEGF therapy, implying a synergistic potential when losartan is combined with vascular‐targeting agents [62]. The vascular effects of losartan can be augmented through strategic drug design or involve crosstalk with other pathways. For instance, in glioblastoma, a covalent quercetin–losartan conjugate markedly simplified tumour vasculature where losartan monotherapy showed limited efficacy [84]. Meanwhile, in cholangiocarcinoma, AT1R blockade attenuated angiogenesis by suppressing oncogenic YAP/TEAD signalling, revealing Hippo‐pathway crosstalk that may shape tumour‐specific vascular phenotypes [85].

Collectively, these data indicate that losartan can (i) normalise dysfunctional vessels and improve perfusion in tumours where stromal compression is dominant, (ii) stabilise microvascular architecture in tumours with pericyte‐related defects and (iii) act synergistically with targeted agents (e.g., VEGF inhibitors, multi‐kinase inhibitors) to overcome resistance—while the relative contribution of each mechanism varies by tumour histology and microenvironmental context [13, 42, 62, 64, 80, 81, 84, 85]. The vascular normalisation induced by anti‐angiogenic agents is known to be transient, confined to a critical ‘normalisation window’ that represents a therapeutic opportunity for improved drug penetration [79]. Given that losartan treatment produces a similar phenotype of vessel stabilisation and improved perfusion, its benefits are also likely time‐dependent, and the challenge of defining the optimal timing, dosage and sequence of losartan‐based regimens is further compounded by the stark heterogeneity of its mechanisms [13]. As evidenced by the spectrum of responses—from stromal decompression in PDAC to pericyte stabilisation in schwannomas—it is clear that a universal dosing strategy may be ineffective, and the development of predictive biomarkers will be paramount to matching the right tumour type with the appropriate losartan‐based combination in future clinical studies.

This vascular remodelling is closely linked to the ECM‐targeting effects described previously. By reducing collagen I synthesis/deposition, HA accumulation and matrix stiffness, losartan alleviates solid stress on tumour vasculature and restores perfusion independently of VEGF inhibition [18]. Collectively, these tumour‐type–specific effects indicate that losartan modulates angiogenesis by simultaneously targeting both the Ang II/VEGF and YAP/TAZ pathways. By correcting vascular abnormalities and enhancing drug delivery, losartan holds the potential to broadly improve the efficacy of diverse anti‐tumour therapies.

## 4 Losartan‐Enhanced Nanodelivery: Preconditioning and Co‐Delivery Strategies

### 4.1 Losartan Preconditioning to Enhance Nanotherapeutic Delivery

**FIGURE 3:**
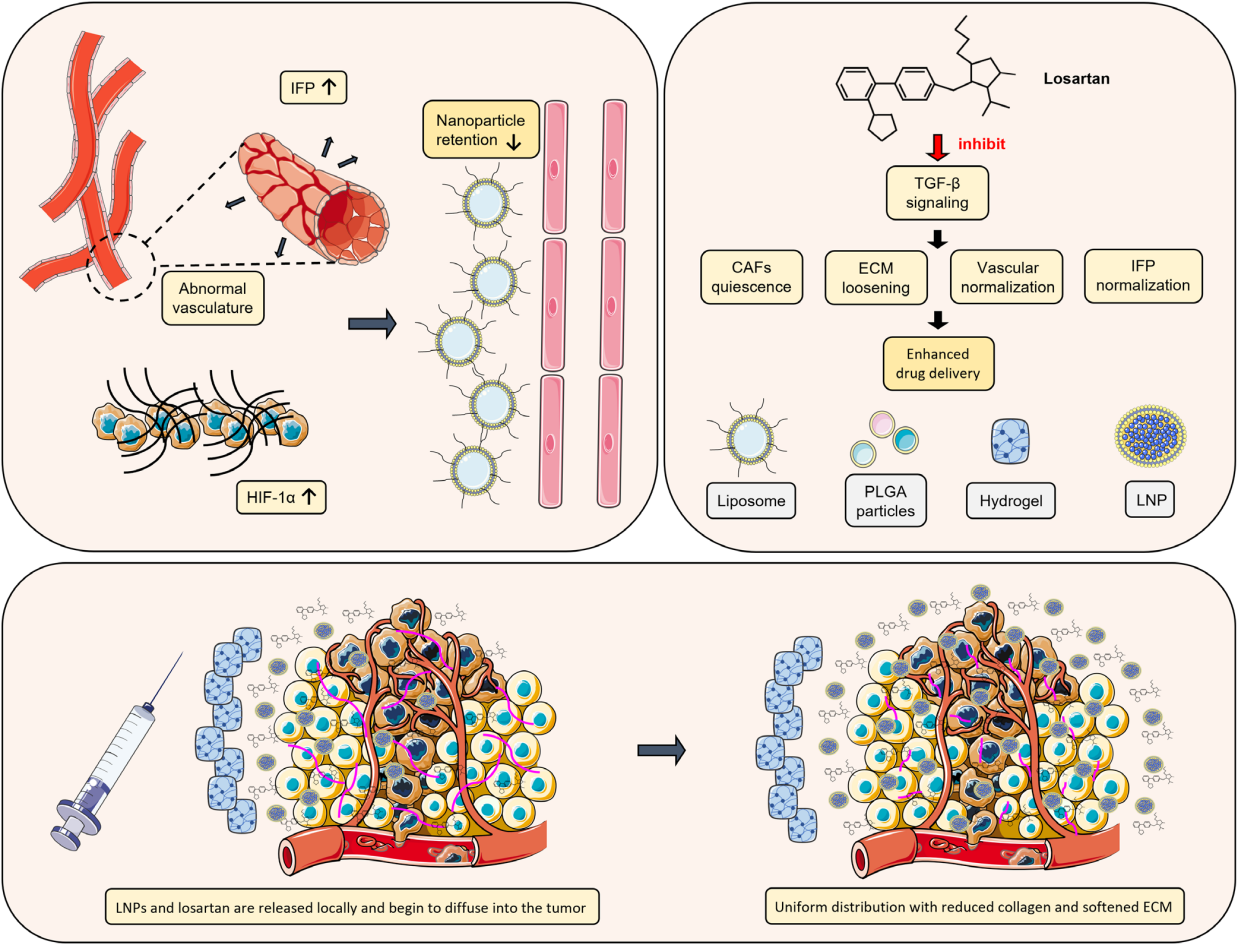
Losartan enhanced nanomedicine delivery via ECM remodelling potentially involving TGF‐β signalling. The schematic representations of drugs, cells and blood vessels in this figure were adapted from SMART Servier Medical Art, licensed under CC BY 3.0.

The efficacy of conventional chemotherapy is often compromised by severe off‐target toxicities, including hematologic, renal and neurological adverse effects. Although intratumoral injection offers localised delivery, it frequently causes drug leakage into adjacent normal tissues, leading to collateral damage. Nanomedicine platforms—such as liposomes, polymeric nanoparticles and hydrogels—have been developed to improve tumour‐specific drug accumulation and minimise systemic exposure. However, efficient delivery of nanotherapeutics remains strongly constrained by the TME, which is characterised by a dense ECM, abnormal vasculature and elevated IFP [20, 62, 86]. The fibrotic stroma restricts nanoparticle diffusion, abnormal vessels impair perfusion and elevated IFP generates a pressure gradient opposing intratumoral transport (Figure 3).

Losartan alleviates stromal density and fibrosis, resulting in vascular decompression and improved perfusion in fibrotic tumours such as PDAC, thereby enhancing intratumoral drug delivery [13]. Mechanistically, losartan downregulates profibrotic mediators including CTGF and α‐SMA, leading to substantial reductions in collagen I deposition and ECM stiffness. For example, in pancreatic (L3.6pl) and other solid tumour models, losartan treatment reduced collagen I levels by 42% to 50% [18]. Notably, CTGF is a transcriptional target of both TGF‐β signalling and the YAP/TAZ mechano‐transduction cascade. These ECM modifications markedly improve the intratumoral distribution and retention of nanomedicines [18].

In preclinical models, losartan preconditioning significantly enhances the penetration and accumulation of nanotherapeutics. This effect was closely associated with inhibition of collagen I synthesis and stromal remodelling [18]. For instance, in sarcoma models, losartan increased the intratumoral distribution of intratumorally injected 100‐nm nanoparticles by 1.5 to 4‐fold and systemically administered ones by twofold. Concurrently, it reduced tumour collagen I levels by 20% to 50% across different models [18]. In a pancreatic cancer patient‐derived xenograft (PDX) model, preconditioning with a nano‐formulated losartan normalised tumour vasculature and improved perfusion, resulting in 1.8‐fold higher accumulation of SN38‐loaded nanoparticles compared to the saline control [20]. Similarly, inhalation delivery of ARBs in lung cancer models markedly enhanced nanoparticle accumulation; specifically, losartan and telmisartan increased intratumoral nanoparticle distribution by 5.3‐fold and 14.3‐fold, respectively, compared to the control [86]. Beyond pharmacological approaches, physical modalities like photothermal (PTT) and photodynamic (PDT) therapies offer a distinct strategy for ECM modulation. Platforms such as porphyrin‐based covalent organic frameworks (COFs) or carbon nanomaterials ablate physical barriers through localised hyperthermia or ROS burst, which can disrupt dense collagen networks [87]. This physical approach contrasts with the biochemical, TGF‐β‐mediated pathway inhibition employed by losartan.

Importantly, this mechanistic dichotomy provides a rationale for combination therapy. Emerging preclinical evidence directly demonstrates the synergistic potential of combining losartan's biochemical modulation with physical energy modalities. In neuroblastoma models, a combination regimen of losartan, platinum nanoparticles (PtNPs) and PTT not only induced immunogenic cell death (ICD) but also effectively suppressed HIF‐1α expression—a key mediator of treatment resistance. Critically, this triple combination exhibited no significant systemic toxicity (as evidenced by stable body weight and normal organ histology) at the optimised dose, thereby successfully expanding the therapeutic window through nano‐delivery [46]. In breast cancer models, losartan preconditioning significantly depleted tumour collagen content (by 53%), which consequently enhanced the deep tumour penetration of a chlorin e6 (Ce6)‐based photosensitiser. This dramatic ECM remodelling led to a superior antitumor efficacy, with the combination of losartan and Ce6‐mediated photodynamic therapy achieving a tumour volume suppression rate of 82% [88].

While these combination strategies have shown compelling results, several translational challenges must be acknowledged. The necessity for preconditioning complicates clinical scheduling, and the heterogeneous stromal composition across tumours suggests that the enhancement in drug delivery will likely be patient‐specific. Addressing these hurdles of timing and patient stratification will be crucial. Meanwhile, investigating the deepest mechanistic crosstalk—such as how losartan‐induced vascular normalisation affects thermal distribution during PTT or oxygen availability for PDT—and engineering next‐generation, intelligent nanoplatforms that seamlessly integrate these dual modalities represent high‐value directions for future research [87, 89].

### 4.2 Losartan‐Integrated Co‐Delivery Nanoplatforms

The direct incorporation of losartan into nanocarrier systems has emerged as a powerful strategy to enhance its bioavailability, tumour‐targeting efficiency and pharmacokinetic profile. This approach enables localised drug release within the TME, significantly reducing systemic toxicity while synergizing with co‐delivered therapeutic agents (Figure 3) [20, 90].

#### 4.2.1 Hydrogel‐Based Co‐Delivery Systems

In a subcutaneous breast cancer model, peritumoral implantation of a hydrogel co‐loaded with losartan and nanoparticles increased the intratumoral distribution of nanoparticles by 2.98‐fold compared to nanoparticles alone, through the sustained release of losartan that remodelled the ECM, as evidenced by a 3.56‐fold reduction in collagen [90]. Furthermore, a morphology‐transformable peptide carrier (PL/Pep1) underwent a pH‐responsive transition from nanoparticles to nanofibers after intratumoral injection, forming a stable hydrogel depot that prolonged losartan retention for over 7 days. This system significantly reduced collagen I deposition and synergistically enhanced the penetration of co‐delivered paclitaxel (PTX). The PL/Pep1 treatment group demonstrated the most potent tumour growth inhibition among all groups, with minimal systemic toxicity observed in the breast cancer model [91]. Similarly, a strategy combining a losartan‐loaded hydrogel (LOS@Gel) with oxaliplatin (OX)‐based nanochemotherapy potently reshaped the tumour mechanical microenvironment and reprogrammed the immunosuppressive TME in a resistant TNBC model. This combination resulted in significant tumour suppression and 100% survival over 40 days, alongside increased CD8^+^ T cell infiltration and reduced Tregs [44].

#### 4.2.2 Polymeric Nanoparticle Systems

Poly (lactic‐co‐glycolic acid) (PLGA) nanoparticles are a versatile platform for losartan delivery, leveraging their high encapsulation efficiency and colloidal stability for EPR‐mediated tumour targeting. The capability of the PLGA platform to implement complex therapeutic regimens is exemplified in a melanoma model, where a losartan‐loaded formulation served as an effective combination platform, facilitating synchronised drug release, improved intratumoral distribution and multi‐faceted stromal remodelling [92]. The demonstrated ability of such systems to reprogram the tumour microenvironment positions PLGA‐based losartan delivery as a promising strategy with the potential to augment advanced therapies, including immunotherapy.

#### 4.2.3 Cascade‐Responsive and Combinatorial Platforms

Sophisticated nanoplatforms designed for sequential release leverage losartan to prime the TME before activating subsequent therapy. One such strategy sequentially releases losartan to degrade the ECM and enhance penetration, followed by the release of chemo‐immunotherapeutic agents. This approach effectively disrupts the CAF‐CD8^+^ T cell immunosuppressive axis, depletes immunosuppressive Tregs and robustly activates antitumor immunity [93]. In parallel, other cascade‐responsive systems disrupt immune‐fibroblast crosstalk to potentiate mechano‐immunotherapy [94]. Similarly, liposomal carriers have been developed as combinatorial platforms. For instance, multifunctional liposomes co‐loaded with losartan and the TLR7/8 agonist resiquimod (R848) markedly enhanced dendritic cell (DC) activation, increasing the population of CD80/CD86 double‐positive DCs in tumour‐draining lymph nodes by 3.5‐fold, and effectively depleted MDSCs in a breast cancer model [95].

#### 4.2.4 Platforms Integrating Physical Modalities and Immunotherapy

Losartan‐integrated nanocarriers effectively synergise with energy‐based therapies to potentiate immunomodulation. For instance, an ultrasound (US)‐responsive nanosensitizer co‐delivering losartan and polyethylenimine (PEI) demonstrated robust TME remodelling capabilities, significantly depleting collagen and enhancing drug permeability. This remodelling, combined with the reprogramming of tumour‐associated macrophages from an M2 to an M1 phenotype, transformed immunologically ‘cold’ tumours and markedly enhanced the efficacy of anti‐PD‐L1 therapy, achieving a 90% tumour growth inhibition rate—1.7‐fold higher than anti‐PD‐L1 therapy alone [96]. Similarly, porphyrin‐based COFs encapsulated with hyaluronic acid and loaded with losartan have been developed for targeted combination therapy. This platform achieved efficient ECM disruption and generated substantial ROS under NIR irradiation, which significantly enhanced the deep‐tumour penetration and efficacy of sequentially administered chemotherapeutic agents [97]. In another approach, a PTT‐induced feedback carbon nano‐system functionalised with losartan and the targeting peptide iRGD was engineered for ECM remodelling in breast cancer. This system achieved a substantial reduction in collagen deposition and exhibited remarkable tumour growth inhibition through synergistic PTT‐ECM modulation [98].

Additionally, multidrug co‐delivery nano‐systems incorporating losartan with immunomodulators have demonstrated potent activity in disrupting the tumour immune microenvironment. A responsive cocktail nano‐strategy co‐delivering losartan, a STING agonist and a PD‐L1 inhibitor effectively broke the immune‐excluded state in TNBC. This approach reduced the proportion of immunosuppressive Tregs while significantly enhancing cytotoxic T lymphocyte infiltration and dendritic cell maturation, ultimately reversing the immunosuppressive microenvironment and achieving potent antitumor efficacy [93].

#### 4.2.5 Summary of Platform Diversity and Efficacy

A recent comprehensive review positions losartan as a promising mechano‐therapeutic adjuvant for remodelling the breast tumour microenvironment, highlighting its broad potential in oncology nanomedicine [38]. While the diversity of losartan‐integrated platforms is impressive, their sophisticated designs also introduce significant challenges for scalable manufacturing and reproducible quality control, which must be addressed for clinical translation. This potential is being realised through its successful integration into a wide spectrum of delivery platforms—including liposomes, polymeric nanoparticles, injectable hydrogels and photothermal nanoplatforms, which leverage its ability to prime the TME for enhanced therapy [44, 91, 92, 96, 97, 98]. The strategies for combining losartan extend from these sophisticated, integrated co‐delivery systems to the simpler yet effective approach of co‐administration. Notably, the co‐administration of losartan was shown to facilitate the penetration of TRAIL mRNA‐loaded lipid nanoparticles and inhibit tumour progression in colon cancer models [99]. Collectively, these findings underscore losartan's exceptional compatibility with diverse nanocarriers and its versatility in combination therapies [20, 90]. Beyond enhancing pharmacokinetics, these losartan‐based systems confer advanced tumour‐adaptive functions—such as TME‐responsive release, immune activation and ECM remodelling—which are pivotal for overcoming therapeutic resistance. Importantly, the therapeutic potential of these platforms is preliminarily supported by their favourable safety profiles; multiple preclinical studies have reported that losartan‐loaded nanoplatforms are well‐tolerated, with no significant systemic toxicity observed at therapeutic doses [46, 88].

## 5 Direct Antitumor and Immunomodulatory Effect

### 5.1 Anti‐Proliferative and Pro‐Apoptotic Activities

Ang II promotes tumour progression by activating the AT1R signalling axis, which stimulates multiple oncogenic pathways, including PI3K/Akt, Raf/MEK/ERK and Wnt/β‐catenin. As a selective AT1R antagonist, losartan interferes with these downstream cascades, exhibiting anti‐proliferative and pro‐apoptotic effects across several tumour types. However, its therapeutic efficacy appears highly dependent on tumour histology, molecular context and microenvironmental features.

The PI3K/Akt pathway is a major effector downstream of AT1R. In NK/T‐cell lymphoma, Ang II‐stimulated proliferation is effectively suppressed by losartan [100]. Similarly, Ang II/AT1R signalling promotes breast cancer cell survival via the PI3‐kinase/Akt pathway, an effect reversible by losartan [101]. In gastric cancer, where Ang II promotes progression, losartan directly inhibits cell proliferation and induces apoptosis [39, 102]. However, this anti‐proliferative effect was observed at a high concentration (IC_50_ ≈ 3000 μM) that far exceeds pharmacologically achievable levels. Furthermore, in vivo studies demonstrate that losartan suppresses tumour progression in xenograft models, at least in part by reducing lymphatic microvessel density (LMVD) and VEGF‐C expression [103]. A notable example of its potency comes from nasopharyngeal carcinoma models, where losartan monotherapy achieved a marked tumour growth inhibition [104]. This indirect, upstream targeting approach differentiates losartan from direct PI3K/Akt inhibitors like alpelisib, which is FDA‐approved for PIK3CA‐mutated breast cancer, and may lead to distinct efficacy and resistance profiles [105].

Beyond the PI3K/Akt axis, losartan exhibits efficacy against other oncogenic pathways. In HCC, it inhibits the central Raf/MEK/ERK proliferative axis and the pro‐inflammatory PKC/NF‐κB pathway, the latter by preventing NF‐κB p65 nuclear translocation [51]. This multi‐targeted action in vivo—evidenced by significantly reduced serum AFP levels and prolonged survival—differs from direct MEK inhibitors like trametinib by simultaneously normalising the inflammatory microenvironment via the suppression of the NF‐κB pathway, leading to downstream decreases in TNF‐α, TGF‐β, VEGF and MMP‐2 [51, 106].

Losartan also modulates the Hippo‐YAP pathway. In ovarian cancer, it restores Hippo pathway integrity and attenuates YAP‐driven oncogenic signalling [107]. Notably, under genotoxic stress conditions, YAP may shift roles and promote apoptosis through p73‐dependent mechanisms, suggesting that losartan's modulation of YAP signalling is context‐dependent [108]. This functional duality of YAP presents a layer of complexity not typically associated with conventional, unidirectional targeted therapies. The efficacy of losartan is highly context‐dependent, varying not only between cancer types but also in its primary mechanism of action—ranging from direct tumour cell cytotoxicity to microenvironment modulation. For instance, in NF2‐associated vestibular schwannoma, losartan fails to directly suppress tumour cell proliferation but achieves significant clinical benefits by preventing hearing loss and enhancing radiation efficacy through TME normalisation [81]. This variability, coupled with clinical considerations such as hypotension, underscores the urgent need for predictive biomarkers. In summary, losartan exerts anti‐tumour effects by modulating multiple AT1R‐associated signalling pathways. Its highly tumour‐specific and mechanism‐divergent efficacy highlights the necessity for rigorous biomarker‐driven patient selection and rational combination strategies.

### 5.2 Anti‐Invasive and Anti‐Metastatic Effects

Tumour metastasis is a complex, multistep process and remains the primary cause of cancer‐related mortality. This process is profoundly influenced by tumour location, vascular and lymphatic architecture and microenvironmental factors. Hypoxia, a hallmark of aggressive tumours, stabilises HIF‐1α, which upregulates LOX, a critical mediator of the pre‐metastatic niche [109]. LOX enhances collagen I crosslinking and ECM remodelling, increasing ECM stiffness. This stiffened matrix promotes integrin‐mediated mechano‐transduction, facilitating tumour cell migration and invasion [110, 111]. Losartan inhibits metastatic progression through multiple mechanisms. By blocking AT1R signalling, it reduces collagen deposition and LOX activity, thereby alleviating ECM stiffness [41]. In breast cancer, Ang II/AT1R promotes EMT and lymph node metastasis via CXCR4/SDF‐1α, a process integral to lymphangiogenesis and metastatic spread [112, 113]. Losartan may target similar pathways in other cancers, and preliminary evidence from a phase II trial in pancreatic cancer is emerging, pending confirmation in larger randomised studies [40].

In murine NSCLC models, losartan restores epithelial marker E‐cadherin and suppresses mesenchymal marker vimentin. Furthermore, preclinical evidence suggests that angiotensin system inhibitors like losartan can reverse the epithelial‐mesenchymal transition phenotype and potentiate the efficacy of cisplatin, leading to reduced tumour growth [19]. This synergistic effect is corroborated by retrospective clinical analyses. For instance, a retrospective cohort study reported a significantly longer median survival of 11.7 months in patients receiving ACEIs/ARBs compared to 8.6 months in controls (an increase of ~36%), although the findings may be influenced by incomplete adjustment for confounders [114, 115, 116, 117, 118]. In a murine breast cancer (4T1‐luc) experimental lung metastasis model, treatment with losartan (60 mg/kg) mediated AT1R‐independent anti‐metastatic effects, likely through inhibiting CCR2‐dependent monocyte recruitment [119]. It noncompetitively inhibits ERK1/2 phosphorylation downstream of the CCL2/CCR2 axis—a key pathway in recruiting inflammatory monocytes to facilitate metastasis [120]. By reducing CCR2 surface expression on monocytes and limiting their recruitment to metastatic sites—a critical step in the formation of metastasis‐associated macrophages—losartan drastically suppresses pulmonary metastatic colonisation [119]. This unique, off‐target mechanism positions losartan differently from selective CCR2 inhibitors developed specifically for cancer therapy [121].

Beyond hematogenous spread, lymphatic metastasis is particularly relevant in ovarian cancers, where diaphragmatic lymphatic dysfunction contributes to ascites. In preclinical models, losartan reduces ascites not by inhibiting VEGF, but by depleting tumour ECM to relieve solid stress and decompress diaphragmatic lymphatic vessels, thereby improving fluid drainage. This enhancement of drug delivery synergises with chemotherapy, a finding supported by clinical data showing that ovarian cancer patients receiving ARBs/ACEI had a significantly longer median overall survival (63 months) than those on other antihypertensives (33 months) [64]. Furthermore, reprogramming the TME with angiotensin blockers can synergise with cancer immunotherapy [22].

In summary, losartan exerts multifaceted anti‐metastatic effects by modulating EMT, inhibiting pro‐metastatic macrophage recruitment and restoring lymphatic function. However, the translational path faces hurdles, including the need to achieve effective anti‐metastatic concentrations in patients and to identify predictive biomarkers. Furthermore, the clinical development of losartan in oncology must be contextualised within ongoing safety discussions. A meta‐analysis of randomised controlled trials by Sipahi et al. reported a modestly increased risk of new cancer diagnosis with ARBs (Risk ratio = 1.08), prompting further scrutiny by regulatory agencies [122]. Consequently, dedicated trials are necessary not only to confirm its anti‐metastatic efficacy but also to fully define its risk–benefit profile in cancer patients.

### 5.3 Overcoming TME Barriers to Enhance Chemo‐ and Radiotherapy

Tumour vasculature is often structurally abnormal and functionally inefficient, leading to regions of intermittent hypoxia and reoxygenation that compromise the efficacy of both radiotherapy and chemotherapy [123, 124]. Chemotherapy is further limited by non‐specific cytotoxicity, dose‐limiting toxicities such as platinum‐induced neurotoxicity and anthracycline‐associated cardiotoxicity, and the frequent emergence of drug resistance. In addition, radiotherapy can induce fibrosis by upregulating collagen I and TGF‐β expression, activating the TGF‐β/Smad2 pathway and increasing ROS production [125, 126, 127].

These barriers contribute to therapy resistance across a spectrum of solid tumours. For instance, radiotherapy to the head/brain can cause late ototoxicity [128]. In NF2 schwannoma preclinical models, losartan ameliorated tumour‐associated hearing loss and augmented radiation efficacy, suggesting a potential therapeutic avenue for further clinical investigation [81]. Anti‐angiogenic therapy with agents such as bevacizumab can result in hypertension and proteinuria [129, 130, 131]. Osteosarcoma remains a major therapeutic challenge in humans, with limited effective treatments. In a canine comparative model, losartan combined with a kinase inhibitor (toceranib) achieved a 50% clinical benefit rate in dogs with metastatic disease and showed survival improvement [132]. While promising, it remains to be tested whether similar effects translate to human patients. In TNBC, stromal barriers restrict drug penetration and resistance to targeted therapies frequently develops [133]. Glioblastoma is characterised by blood–brain barrier disruption and cerebral edema [134, 135, 136]. High‐grade serous ovarian cancer and HCC face challenges including TKI resistance and treatment‐related adverse effects, such as lenvatinib‐induced hypertension and sorafenib‐associated hand‐foot syndrome [64, 137, 138, 139, 140].

Losartan has been investigated as a TME‐modulating agent with the potential to overcome these treatment barriers. By decompressing tumour blood vessels through the reduction of solid stress, losartan significantly improves vascular perfusion, increasing the fraction of perfused vessels from ~21%–23% to ~43%–45% (approximately a twofold increase) in desmoplastic tumour models. This enhanced perfusion, in turn, boosts the delivery of chemotherapeutic agents, such as increasing 5‐fluorouracil (5‐FU) accumulation in pancreatic tumours by 74% [13]. In preclinical HCC models, losartan sensitised tumour cells to lenvatinib such that low‐dose lenvatinib plus losartan produced antitumor effects comparable to higher‐dose regimens in xenografts; whether this strategy reduces clinical toxicity remains to be validated in patients [42]. Furthermore, its potential application in desmoid tumours suggests a broader role in modifying fibrotic tumour environments where surgical intervention carries significant risks [141]. This ‘stromal normalisation’ approach contrasts critically with failed strategies that aggressively deplete cancer‐associated fibroblasts, which have been shown to exacerbate disease progression and impair survival, highlighting the importance of modulating rather than destroying the TME [72].

Beyond its direct impact on the physical TME, losartan's benefits extend to mitigating specific treatment‐induced toxicities. A key example is its protective effect against chemotherapy‐induced peripheral neuropathy (CIPN), a common and often dose‐limiting side effect. Preclinical studies robustly demonstrate that losartan attenuates neuroinflammation and mechanical hyperalgesia in models of paclitaxel‐induced neuropathy [142]. The mechanism is linked to the direct inhibition of AT1R within the dorsal root ganglia, leading to a reduction in pro‐inflammatory cytokines and thereby alleviating pain hypersensitivity [143]. The translational potential of this approach is underscored by preliminary clinical observations. In the OncoToxSRA retrospective cohort, the use of RAS inhibitors was suggested to be associated with a significantly lower incidence of CIPN in patients receiving neurotoxic chemotherapy [144]. By mitigating this major chemotherapy‐related toxicity, losartan may improve patients' quality of life and potentially enable the administration of more complete treatment cycles. Similar microenvironment‐targeting strategies, such as the use of curcumin to overcome multidrug resistance in lung adenocarcinoma, remain limited by poor bioavailability [145, 146].

Losartan, by remodelling the ECM and improving vascular function, represents a potential adjunctive approach to enhance treatment delivery and efficacy. The promising preclinical results demonstrating improved perfusion and chemosensitisation now necessitate clinical validation. Key to this effort will be resolving the disconnect between the high doses required for TME modulation in models and the exposures achieved with conventional antihypertensive regimens. The development of companion biomarkers is equally vital to identify patients with fibrotic, perfusion‐compromised tumours who are most likely to benefit from this stromal‐normalising strategy.

### 5.4 Tumour Immune Microenvironment Remodelling

Tumour fibrosis and hypoxia contribute to an immunosuppressive TME, characterised by limited infiltration of effector immune cells and impaired anti‐tumour responses [147]. This environment promotes immunosuppressive populations—including M2‐polarised TAMs, MDSCs and Tregs—which secrete cytokines such as IL‐6, IL‐10 and TGF‐β to drive tumour progression and suppress effector CD8^+^ T cells, NK cells and DCs [148, 149].

In poorly immunogenic tumours such as TNBC, immune checkpoint inhibitors (ICIs) often fail due to the polarisation of M1 macrophages toward the M2 phenotype, contributing to T cell exhaustion and immune escape [148, 150, 151]. Moreover, in immune‐excluded ‘cold’ tumours with dense fibrotic stroma and poor T cell infiltration, the efficacy of ICIs remains limited. ARBs such as losartan have been shown to disrupt fibrotic barriers, enhance T cell penetration and reprogram the immunosuppressive microenvironment, converting immune ‘cold’ tumours into ‘hot’ ones and synergizing with anti‐PD‐1 therapy [45]. This approach breaks down physical and immunological barriers in armoured tumours, facilitating enhanced immune cell access [45]. Through AT1R blockade, losartan remodels this suppressive milieu. It promotes the repolarization of M2 TAMs toward a pro‐inflammatory M1 phenotype, reduces the accumulation of Tregs and MDSCs, enhances intratumoral infiltration of CD8^+^ T cells and promotes DCs maturation [21, 22, 134]. In neurofibromatosis models, losartan also alleviates immune suppression by inhibiting IL‐6/STAT3 in TAMs through AT1R blockade, correlating with clinical benefits like hearing restoration [81].

Beyond its local immunosuppressive effects, losartan may mitigate systemic manifestations such as cancer cachexia, as Ang II is known to promote muscle proteolysis. Additionally, preclinical studies suggest a protective role against cancer‐associated cardiac dysfunction [152]. While these potential benefits are promising, direct evidence from cachexia models remains to be established. Separately, in pancreatic cancer, losartan has been shown to reduce immunosuppressive genes and FOXP3^+^ cancer cells, underscoring its capacity to reverse key mechanisms of therapy resistance [40].

Despite compelling preclinical evidence across multiple cancer types, the clinical translation of losartan's immunomodulatory effects requires cautious optimism [64]. A clinical study in metastatic pancreatic cancer found that concurrent losartan use was not associated with improved overall survival in patients receiving chemotherapy, underscoring the discordance between robust preclinical models and complex human disease [153]. Future work must define the patient populations and therapeutic contexts in which losartan can deliver meaningful clinical benefit.

### 5.5 Limitations and Future Directions

Despite promising preclinical findings, clinical evidence supporting the anti‐tumour efficacy of losartan remains limited and somewhat ambiguous. For instance, while some retrospective analyses have hinted at potential benefits in specific subgroups (e.g., patients receiving FOLFIRINOX), the overall data from cohorts like that of Kasi et al. have not demonstrated a significant survival advantage. Consequently, the most compelling human data are currently confined to pancreatic cancer and cholangiocarcinoma, with robust evidence in other major tumour types still lacking [153, 154]. A critical translational gap lies in the fact that the anti‐tumour effects observed in preclinical models often require drug exposures substantially higher than those achieved with standard antihypertensive dosing [13]. This underscores the pressing need for optimised delivery strategies and, crucially, biomarker‐guided patient selection.

The fundamental association between losartan use and cancer risk remains a subject of debate. Large‐scale meta‐analyses of randomised controlled trials have yielded conflicting conclusions, with some suggesting a potential class‐wide increase in cancer risk for ARBs, while others find no significant association [122, 155, 156, 157]. These discrepancies may arise from differences in study populations, follow‐up duration and confounding factors (e.g., smoking, obesity), highlighting the challenge of interpreting epidemiological data for drug repurposing [122, 158].

Positioning losartan within the broader pharmacological landscape of TME‐targeting agents reveals both its unique value and its limitations. While potent anti‐fibrotic agents like pirfenidone and the HA‐degrading enzyme PEGPH20 have shown promise in preclinical models and late‐stage clinical trials, their development has been hampered by toxicity or limited efficacy in unselected populations [159, 160]. In contrast, losartan acts upstream via the RAS/TGF‐β axis to concurrently remodel both the ECM and vascular networks, offering a broader mechanism of action [13, 18]. This multi‐faceted activity was demonstrated in an ovarian cancer model, where losartan normalised the tumour stroma, enhanced chemotherapy efficacy and reduced ascites [64]. However, its anti‐fibrotic potency may be moderate compared to these dedicated agents, potentially necessitating higher doses or advanced delivery systems for maximal effect. Future studies must therefore prioritise biomarker‐driven approaches. Biomarkers reflecting ECM remodelling (e.g., HA levels, collagen density), TGF‐β pathway activation or an immunosuppressive TME could identify patients most likely to benefit. These considerations are highly relevant for ongoing and completed clinical trials evaluating losartan combinations. For instance, the phase II study (NCT03563248) of losartan with nivolumab in resectable pancreatic cancer aims to assess stromal and immune remodelling. Separately, the landmark study by Murphy et al. (NCT01821729) established the feasibility of adding losartan to FOLFIRINOX and chemoradiation, which was subsequently shown by Boucher et al. to be associated with a reduction in immunosuppressive Tregs and FOXP3^+^ cancer cells [25, 40].

Preliminary findings from these trials indicate that losartan‐mediated stromal remodelling may improve vascular perfusion and intratumoral drug penetration [13, 18]. However, the available clinical data remain largely preliminary and conclusive evidence for a survival benefit is still awaited. Furthermore, inter‐individual variation in losartan pharmacokinetics due to genetic polymorphisms (e.g., in CYP2C9) may influence drug exposure and response, adding another layer of complexity to its clinical application [29]. Rigorous trials that directly compare losartan to other stromal‐targeting agents and evaluate its integration into novel combination regimens across various fibrotic tumours are essential to define its unique therapeutic niche.

## 6 Conclusion

Losartan repurposing in oncology capitalises on its unique ability to modulate multiple components of the TME. Beyond its well‐established role in reducing stromal density and normalising tumour vasculature, losartan demonstrates significant potential in reshaping the immune landscape and suppressing metastatic dissemination. These multifaceted TME‐modulating mechanisms, together with losartan's long clinical history and generally favourable safety profile in cardiometabolic indications, provide a biologically plausible rationale for its evaluation as an adjunct in oncology—although definitive randomised clinical evidence in cancer remains limited. However, translating these preclinical findings into clinical benefit remains challenging. The current evidence base is predominantly concentrated in three areas: robust clinical‐translational evidence in PDAC (exemplified by the phase II trial of Murphy et al. and subsequent analysis by Boucher et al.); supportive retrospective clinical analyses in cholangiocarcinoma (e.g., Li et al.); and compelling preclinical data in breast cancer models (e.g., from Chauhan and Diop‐Frimpong). This landscape reveals a critical gap in our understanding of its efficacy across other malignancies. A paramount challenge is the preclinical‐to‐clinical dose disconnect, wherein antitumor efficacy often requires exposures surpassing standard antihypertensive dosing.

To bridge this translational gap, future efforts must be strategically directed. Priority should be given to stroma‐rich tumours where TME remodelling is most relevant. Research must refine rational combination strategies with chemotherapy, radiotherapy and particularly immunotherapy, and pioneer advanced delivery systems—such as nanoparticle formulations—to achieve effective intratumoral drug concentrations. Ultimately, the successful repurposing of losartan hinges on rigorously designed, biomarker‐driven clinical trials that are essential to definitively establish its efficacy, optimise its integration into multimodal regimens, and secure its niche in the evolving landscape of precision cancer therapy.

## Author Contributions

**Han Wang:** writing – original draft (lead), writing – review and editing (lead). **Shuang Yuan:** writing – review and editing (equal). **Hongjing Wang:** conceptualization (equal), writing – review and editing (equal).

## Funding

The authors have nothing to report.

## Ethics Statement

The authors have nothing to report.

## Consent

The authors have nothing to report.

## Conflicts of Interest

The authors declare no conflicts of interest.

## Data Availability

All data generated or analysed during this study are included in this published article.
